# Committees and Justice

**Published:** 1907-11-02

**Authors:** 


### Nursing Administration.

### COMMITTEES AND JUSTICE.

Nothing could more forcibly illustrate the defects of the system under which large groups of institutions are subjected to regulations made for them *en bloc* by committees unacquainted with the details of their work than the recent decision of the Metropolitan Asylums Board Committee to lower the status of all the matrons in their service. It is very important to realise that the danger is one to which all rate-supported institutions are exposed. The principle of public support carries with it the principle of public control, a principle which practically appears to mean risk of high-handed and light-hearted interference with long-established and valuable methods by anyone who has the courage of his opinions, and can induce a sufficient number of the committee to follow his lead. Let it be supposed that the board of any voluntary hospital, composed of persons imbued with a true sense of their responsibility towards the establishment, were to have before them a draft order altering the appointments of a certain class of officer, and lessening the authority which had long been associated with the post. The ordinary course followed would be to defer the consideration of the proposal until the fullest inquiry could be made into the manner in which the proposed alteration would affect persons holding the post, and those who worked in association with them. It would be the duty of the secretary to prepare a report stating what reasons there appeared to be for making the change, and the persons most nearly concerned would certainly be consulted, and their judgment allowed its full weight.

It is not too much to say that these careful investigations would have been undertaken as a matter of course by any committee able to comprehend the extent of its responsibilities, even if the proposed changes were only contemplated for one institution, and for subordinate officers. What a different method of procedure do we note in the newly-appointed committee of the Metropolitan Asylums Board. Hasty discussion, no delay, no consultation with those most nearly concerned, no evidence adduced of abuses, no consensus of opinion from those working in the institutions. Here are some two thousand institutions, according to the reckoning of Sir R. M. Hensley himself, recklessly interfered with at the instance of a group of people who have no conception of the magnitude of the work they are wrecking.

It is remarkable that the Chairman at the last meeting of the Board exhibited entire incapacity to grasp the point at issue. While declaring in one sentence that the position of the matrons was unaffected by the new draft order, he went on to state that the order expressly provided that the chief officer of every establishment should be the head of the establishment and have supreme control, subject to the regulations of the managers, of every officer and servant therein. This, of course, means that every sister, every probationer, every wardmaid or charwoman in these vast establishments receives her orders, performs her duties, and regulates her conduct in full consciousness that the matron, like herself, is a subordinate officer, under the supreme control of another person. The chairman claims credit to the Board for abolishing the classification of officers into "principal" and "subordinate," and affects to ignore the fact that matrons being no longer classed as "principal officers," and being deliberately deprived of supreme control over their staff, are by that very act without any need for nomenclature, debased to the position of "second-class" and "subordinate" officials.

It is almost incredible that the people who voted in favour of this order did so without some impression being conveyed to them that the power hitherto vested in the hands of the matrons had been abused. It is a cruel injustice to brand some of the most experienced matrons of the day, women who have been chosen out of hundreds of candidates for conspicuous talents shown in successful administration in other institutions, with incapacity to wield a power which is accorded to the heads of all training schools as the keynote to success.

It is certain that when these posts come in due course to be filled highly qualified women will hesitate before they place themselves voluntarily in so false a position as is involved in the subordination of the head of the training school to any other person in the institution. We believe the implication which lurks hidden in this obnoxious alteration that friction subsists in any marked degree between the medical and the nursing superintendents, to be absolutely false. So far as our own observation extends, the intercourse between the two persons charged with the duty of maintaining discipline in these huge establishments is marked by courtesy and cordiality. In many respects the maintenance of discipline in these Metropolitan Asylums Board hospitals presents more difficulties than in ordinary hospitals. There is a constant succession in many of them of short-term pupils trained in other establishments. There is a remarkable mixture of classes among the patients. There is a large percentage of patients not seriously ill. There is the necessity for perpetual watchfulness lest infection be borne outside the walls. All these points demand the exercise of unhampered and vigorous administration on the part of the matron, who can only inspire her nurses when her authority in her own department is supreme.

The best fate for this ignorant innovation, carried with much protest from those who were able to understand its mischievous tendencies, among whom we rejoice to see were the medical members of the Board, is to be overruled. And let all and sundry who sigh for the millennium of rate-supported institutions governed by popular vote, take the present lesson to heart.

